# Exploring the origins of frequent tau-PET signal in vermal and adjacent regions

**DOI:** 10.1007/s00259-025-07199-x

**Published:** 2025-03-18

**Authors:** Agnes Kling, Julia Kusche-Palenga, Carla Palleis, Alexander Jäck, Alexander M. Bernhardt, Lukas Frontzkowski, Sebastian N. Roemer, Luna Slemann, Mirlind Zaganjori, Maximilian Scheifele, Lars Paeger, Gérard N. Bischof, Thilo van Eimeren, Alexander Drzezga, Osama Sabri, Michael Rullmann, Henryk Barthel, Johannes Levin, Jochen Herms, Nicolai Franzmeier, Günter Höglinger, Sigrun Roeber, Matthias Brendel, Johannes Gnörich

**Affiliations:** 1 Department of Nuclear Medicine, LMU Hospital, Ludwig Maximilian University of Munich, Munich, Germany; 2 German Center for Neurodegenerative Diseases (DZNE) Munich, Munich, Germany; 3 Department of Neurology, LMU Hospital, Ludwig-Maximilians-University of Munich, Munich, Germany; 4 Institute for Stroke and Dementia Research, LMU University Hospital, LMU Munich, Munich, Germany; 5 Center of Neuropathology and Prion Research, University of Munich, Munich, Germany; 6 Department of Nuclear Medicine, University Hospital Cologne, Cologne, Germany; 7 Institute of Neuroscience and Medicine (INM-2), Research Center Jülich, Jülich, Germany; 8 German Center for Neurodegenerative Diseases (DZNE), Bonn-Cologne, Germany; 9 Munich Cluster for Systems Neurology (SyNergy), Munich, Germany; 10 Department of Nuclear Medicine, University Hospital Leipzig, Leipzig, Germany; 11 Institute of Neuroscience and Physiology, Department of Psychiatry and Neurochemistry, University of Gothenburg, The Sahlgrenska Academy, Mölndal and Gothenburg, Sweden; 12 Department of Neurology, University Hospital Cologne, Cologne, Germany

**Keywords:** Tau-PET, Off-target binding, Vermis, Cerebellum, Meninges, Melanocytes

## Abstract

**Purpose:**

Off-target binding remains a significant challenge in tau-PET neuroimaging. While off-targets including monoamine oxidase enzymes and neuromelanin-containing cells have been identified, recent studies indicated a relevant binding of novel tau tracers to melanin-containing structures. To date, little is known about the effect of melanocytes in the meninges on tracer signals in brain PET data. Thus, we aimed to identify the target structure causal for the frequently observed [^18^F]PI-2620 PET signal in the vermis and adjacent cerebellar regions.

**Methods:**

274 participants underwent dynamic [^18^F]PI-2620 tau-PET: 3/4R-tauopathies (n = 85), 4R-tauopathies (n = 147), tau-negative disease controls (n = 24), and healthy controls (n = 18). Standardized uptake value ratio (SUVR) and kinetic parameters including the distribution volume ratio (DVR), tracer clearance (k2) and relative perfusion (R1), were compared among the cohorts and sexes using the Automated Anatomical Labelling (AAL) atlas. Age and p-Tau levels in cerebrospinal fluid (CSF) were assessed for their relationship with vermal tau-PET signal. Furthermore, we combined autoradiographic and histochemical experiments on post-mortem brain tissue of deceased patients (n = 9).

**Results:**

Male participants revealed higher mean vermal [^18^F]PI-2620 DVR (0.95 ± 0.13; vs. females 0.88 ± 0.10, p < 0.0001). Sex-related differences were most pronounced in the 3/4R-tauopathy cohort (p < 0.0001). Mean SUVR_Ver/Cbl_, k2 and correlation analyses of kinetic parameters did not differ among groups. Histological assessments revealed co-localization of leptomeningeal pigmented cells with strong autoradiography signal spots within the vermal fissures. Tau-related autoradiography signals, age or p-Tau levels did not correlate significantly with tau-PET signals. Iron deposits did not cause relevant autoradiography signals in the vermis.

**Conclusion:**

Leptomeningeal melanocytes are the primary target structure for [^18^F]PI-2620 PET binding in anterior vermis, whereas iron and tau deposits do not contribute significantly.

**Supplementary Information:**

The online version contains supplementary material available at 10.1007/s00259-025-07199-x.

## Introduction

Tauopathies represent a group of neurodegenerative diseases defined by pathological accumulation of non-functional tau protein in the human brain. Tau is responsible for polymerisation and stabilisation of microtubules in neuronal and glial cells. Post-translational modifications lead to functional loss of tau with subsequent dissociation from microtubules, abnormal intracellular deposition and neuronal death [1]. The neuroanatomical distribution of tau deposits and the predominant tau isoform, depending on the number of carboxy-terminal repeat domains, differ among the various tauopathies. Alzheimer’s disease (AD) as the most common form of dementia is characterized by the aggregation of tau isoforms with both 3-repeat (3R) and 4-repeat (4R) binding domains, and corticobasal degeneration (CBD) and progressive supranuclear palsy (PSP) mainly by the 4R isoform [2]. The development of positron emission tomography (PET) with the application of tau-PET tracers has enabled the in-vivo detection of tau deposits [3]. Compared to first-generation tau-PET ligands, novel second-generation tau-PET tracers, particularly [^18^F]PM-PBB3 and [^18^F]PI-2620, have demonstrated high affinity for 3/4R tau isoforms, but also significant binding to 4R tau [4–8]. Although some controversy existed regarding the specificity for 4R tauopathies [6, 9], recent studies have highlighted the potential of [^18^F]PI-2620 to detect 4R tau pathology in primary tauopathies, showing significantly elevated [^18^F]PI-2620 uptake in PSP target regions, which correlated with AT8-positive 4R tau aggregates [4, 8]. In terms of [^18^F]PI-2620, the tracer’s binding characteristics further facilitate discrimination between these tau isoforms by evaluating region-specific distribution volume ratio (DVR), relative perfusion (R1) and tracer tissue clearance (k2) [10, 11].

Nevertheless, interpretation of tau imaging data holds challenges considering off-target binding to molecular structures other than tau protein deposits [12]. Off-target binding remains a key concern for all tau-PET tracers, influencing the reliability of quantifying tau pathology. For instance, although ameliorated compared to first generation agents, [^18^F]MK6240 has been shown to exhibit off-target retention in the meninges and age-related signal in subcortical regions, such as the putamen and pallidum [13]. Similarly, [^18^F]PI-2620 presents distinct off-target binding patterns, particularly in subcortical regions like the midbrain or, to a lesser extent, in the basal ganglia, that may differ in magnitude and spatial distribution from other tracers [14–16]. In addition to monoamine oxidase enzymes [17, 18] and blood products [19], several tau-PET tracers demonstrate off-target binding to structures containing melanin or neuromelanin [9, 17, 19, 20]. Melanin is synthesized in melanocytes, a heterogeneous group of cells derived from neural crest and settled in a range of different organs including skin, eye, ear, heart and nervous system [21]. While previous autoradiography studies demonstrated tau tracer enrichment in tissue samples of the human skin, retinal pigment epithelium, melanoma, and substantia nigra [9, 17, 19, 20], little is known about the impact of melanocytes located in the meninges on molecular imaging data utilizing [^18^F]PI-2620. In particular, enhanced tracer uptake was reported in the anterior vermal and cerebellar regions, a finding observed with several tau-PET tracers and noted in both healthy individuals and patients with distinct neurodegenerative diseases [22–27]. These findings suggest that investigating off-target binding also in non-AD tauopathies, particularly those dominated by 4R isoforms, is essential to optimize tracer utility across diverse tauopathies. Given the close association between cerebellar cortex and meninges, this study aimed to decipher the sources of [^18^F]PI-2620 binding in the vermis. Investigating these patterns is essential for ensuring accurate interpretation of tau-PET scans across diverse tauopathies and for harmonizing quantification approaches across clinical settings. Understanding off-target effects will optimize tracer utility, enhance diagnostic precision, and deepen our understanding of tau pathology.

To this end, we employed dynamic [^18^F]PI-2620 tau-PET scans over 60 min post injection of 256 participants with distinct neurodegenerative diseases and healthy controls (n = 18). Vermal standardized uptake value ratio (SUVR) and tracer kinetics (DVR, k2, R1) were examined among the cohorts and tested for a relationship with sex and age. AT8 immunostaining, iron staining and [^18^F]PI-2620 autoradiography were performed on post-mortem vermal tissue samples of eight cases exhibiting a range of strong to absent in vivo vermal [^18^F]PI-2620 uptake, and one case imaged with a first generation tau radiotracer, exhibiting strong [^18^F]THK-5351 uptake.

## Material and methods

### Study design

In this study, we combined assessments of [^18^F]PI-2620 tau-PET, in vitro autoradiography, tau immunohistochemistry and iron staining using human samples consisting of patients with different tau-positive neurodegenerative diseases and both tau-negative disease controls and healthy controls.

### PET imaging and analysis

#### Subjects

A total of 274 human [^18^F]PI-2620 PET scans were included in our study, acquired between November 2018 and June 2023 at the Department for Nuclear Medicine of the LMU hospital. We only selected patients with a high clinical likelihood (possible or probable to current diagnosis criteria) and healthy controls that received dynamic PET imaging for this study. Participants were classified into four cohorts with clinically diagnosed definite, possible, or probable 3/4R-tauopathies (i.e. AD), 4R-tauopathies (i.e. PSP and CBS), tau-negative disease controls (i.e. Parkinson’s disease, PD; multiple system atrophy, MSA; and dementia with Lewy bodies, DLB), and healthy controls according to current diagnostic criteria [4, 28–30]. Prior to tau-PET imaging, participants underwent comprehensive clinical evaluation and received diagnoses at the Department of Neurology with subsequent follow-up examinations. Additionally, a subset of participants underwent lumbar puncture during their visit to LMU University Hospital, Munich, for cerebrospinal fluid (CSF) analysis. CSF biomarker levels, including Aβ42, Aβ40, and the Aβ42/Aβ40 ratio, were measured using the Innotest ELISA kit (Fujirebio Europe N.V., Belgium). The established cutoff for the Aβ42/Aβ40 ratio was set at > 5.5%, as per standardized diagnostic procedures at the external laboratory. Patients within the 3/4R-tauopathies cohort included individuals with either mild cognitive impairment (MCI) or dementia. These patients had either a positive Aβ42/Aβ40 ratio or exhibited a positive β-amyloid PET ([^18^F]-florbetaben or [^18^F]-flutemetamol), meeting the diagnostic criteria for typical AD [30]. Patients with probable or possible PSP were assessed in accordance with the current diagnostic criteria, with a particular emphasis on closely monitoring disease progression throughout the assessment [28]. In patients with PSP, disease severity was assessed using the PSP Rating Scale, while cognitive impairment severity was evaluated using the Montreal Cognitive Assessment (MoCA) or Mini-Mental State Examination (MMSE) scores. Participants with α-synucleinopathies were clinically assessed using state-of-the-art criteria. These included longitudinal observation of clinical trajectories specifically characterized by parkinsonism in PD, as evaluated with the Unified Parkinson’s Disease Rating Scale (UPDRS), and autonomic failure in conjunction with parkinsonism or ataxia in MSA [31]. Furthermore, for the clinical diagnosis of probable dementia with Lewy bodies (DLB), in addition to cognitive decline, deficits in executive and visuoperceptual functions were assessed in accordance with current guidelines [32]. Healthy controls were obtained from the ActiGlia study and an ongoing Phase 1 trial. All patients and controls who received in vivo PET imaging provided informed written consent. The study was conducted in accordance with the principles of the Declaration of Helsinki, and approval was obtained from the local ethics committee (application numbers 17–569, 19–022). A detailed overview of the study groups is provided in Table 1.

**Table 1:** Demographics

Group	AD	AD-MCI	AD-DEM	4R	CBS	PSP	DC	HC	All
*n*	85	28	57	147	41	106	24	18	274
Age, mean (SD), y	72.7 (± 9.09)	75.1 (± 7.52)	71.6 (± 9.62)	71.2 (± 7.09)	71.1 (± 6.76)	71.2 (± 7.25)	64.5 (± 9.14)	70.1 (± 10.5)	71.0 (± 8.42)
Sex (♀/♂, % female)	44/41 (52%)	14/14 (50%)	30/27 (53%)	59/88 (40%)	15/26 (37%)	44/62 (42%)	8/16 (33%)	10/8 (56%)	121/153 (44%)
CSF Aβ42/40	55	18	37	69	31	38	10	9	n.a
mean % (SD)	4.7 (± 1.4)	4.9 (± 1.5)	4.7 (± 1.3)	7.9 (± 2.0)	7.9 (± 2.1)	7.9 (± 1.9)	6.9 (± 1.5)	8.3 (± 1.4)	n.a
Aβ-PET	40	17	23	48	33	15	2	9	n.a
±	40/0	17/0	23/0	0/48	0/33	0/15	1/1	0/9	n.a
MoCA score, mean (SD)	17.1 (± 5.5)	22.4 (± 4.2)	15.4 (± 4.8)	21.9 (± 4.7)	22.4 (± 5.3)	21.7 (± 4.5)	23.3 (± 4.6)	27.0 (± 1.4)	n.a
MMSE score, mean (SD)	23.6 (± 6.0)	26.7 (± 2.5)	21.3 (± 6.8)	25.6 (± 3.1)	26.8 (± 3.5)	25.0 (± 2.9)	26.7 (± 2.1)	27.4 (± 2.3)	n.a
UPDRS score, mean (SD)	36.1 (± 10.0)	38.0 (± 9.9)	35.5 (± 10.9)	42.2 (± 14.5)	37.6 (± 14.8)	43.9 (± 14.2)	31.9 (± 16.7)	n.a	n.a
PSPRS score, mean (SD)	24.9 (± 7.3)	n.a	25.0 (± 8.0)	33.7 (± 15.5)	22.4 (± 14.6)	37.3 (± 14.1)	28.8 (± 11.4)	n.a	n.a

Abbreviations: *4R* four-repeat tauopathy, *AD* Alzheimer’s disease, *AD-MCI* AD with mild cognitive impairment, *AD-DEM* AD with dementia, *CBS* corticobasal syndrome, *PSP* progressive supranuclear palsy, *DC* disease control, *HC* healthy control, *y* years, *SD* standard deviation, *MoCA* Montreal Cognitive Assessment, *MMSE* Minimal Mental State Examination, *UPDRS* Unified Parkinson’s Disease Rating Scale, *PSPRS *Progressive Supranuclear Palsy Rating Scale, *n.a.* not available

Tau-PET acquisition and preprocessing [^18^F]PI-2620 was synthesized as previously described [33]. The injected dose ranged between 156 and 223 MBq, applied as a slow (10 s) intravenous bolus injection. Positron emission tomography (PET) imaging was performed in a full dynamic setting (scan duration: 0–60 min post injection) using a Siemens Biograph True point 64 PET/CT (Siemens, Erlangen, Germany) or a Siemens mCT (Siemens, Erlangen, Germany). The dynamic brain PET data were acquired in 3-dimensional list-mode over 60 min and reconstructed into a 336 × 336 × 109 matric (voxel size: 1.02 × 1.02 × 2.03 mm^3^) using the built-in ordered subset expectation maximization (OSEM) algorithm with 4 iterations, 21 subsets and a 5 mm Gaussian filter on the Siemens Biograph and with 5 iterations, 24 subsets and a 5 mm Gaussian filter on the Siemens mCT. A low dose CT served for attenuation correction. Frame binning was standardized to 12 × 5 s, 6 × 10 s, 3 × 20 s, 7 × 60 s, 4 × 300 s and 3 × 600 s. All image data were screened for artefacts before including in the study.

#### Tau-PET quantification

Derived from the dynamic PET image acquisition, we reconstructed late-phase [^18^F]PI-2620 tau-PET images for 30–60 min p.i. which were summarized into a single frame after motion correction. Using the Automated Anatomical Labelling (AAL) atlas, the vermis was parcellated in eight regions. To evaluate tau-specific PET binding, DVR were derived from motion-corrected dynamic [^18^F]PI-2620 data using the simplified reference tissue model 2 (SRTM2) as previously described by our group [11], with the inferior cerebellar grey matter serving as the reference region. Additionally, R1 images were generated using SRTM2 to assess neuronal injury and k2 served as surrogate for tracer clearance [34]. SUVR and kinetic parameters (DVR, R1 and k2), were obtained from the lingula (lobules I-II) and the central lobule (lobule III), the lobules where vermal [^18^F]PI-2620 signal was predominantly observed in our clinically acquired PET data and has been repeatedly described in the literature [22–27]. These values were then averaged to a single value for each parameter and patient. Inferior cerebellum (Cbl) served as reference region for calculating SUVR_Ver/Cbl_ and DVR. In the AD cohort, regions of interest (ROIs) were defined for each Braak stage according to the AAL atlas to extract SUVR values as previously described [35]. All above-mentioned parameters were used for statistical analyses.

### Brain tissue samples

#### Subjects

For PET to autopsy analyses, we included nine patients that received either [^18^F]PI-2620 (n = 8) or [^18^F]THK-5351 tau-PET (n = 1) prior to death. Brains were donated to the Munich brain bank and tissue workup was performed by 7st June 2024. Histological evaluation of each case was performed on tissue samples of the anterior lobe of vermis containing lobules I-V and adjacent leptomeninges. The formalin-fixed and paraffin-embedded sections were cut at 4 µm thickness and adjacent slices were used for histochemical and autoradiographic studies. They were collected according to the guidelines of the local ethical committee and usage of the material for this project was additionally approved (application number 19–244). Detailed procedures for immunohistochemistry and autoradiography are provided in the Supplemental Methods and Supplemental Fig. 7.

### Statistical analysis

GraphPad Prism (version 10.1.2, GraphPad Software Inc., San Diego, CA, USA) was used for statistical analysis and illustration of results. Vermal [^18^F]PI-2620 retention (SUVR_Ver/Cbl_) between men and women was compared with Student’s *t*-test. ANOVA tests were carried out to compare vermal tracer uptake (SUVR_Ver/Cbl_) and kinetic parameters (DVR, k2) among individual cohorts. Pearson’s coefficients of correlation were calculated for the correlation analyses with age and phosphorylated tau (p-Tau) levels in CSF, for correlation analysis between signal in vermis and the individual Braak regions, for all correlation analyses of kinetic parameters (DVR, k2, R1) and for correlation analysis between autoradiography ratios and tau-PET signals in autopsy samples. Additionally, a multiple linear regression analysis was performed to assess the contributions of tau abundance and leptomeningeal melanocytes to vermal tau-PET uptake, using AT8 occupancy and hemalum staining (%-area) as independent variables, respectively. A significance level of p < 0.05 was applied in all analyses.

## Results

### *Vermal lobules I-III frequently exhibit strong [*^*18*^*F]PI-2620 tau-PET signals*

**Fig. 1:**
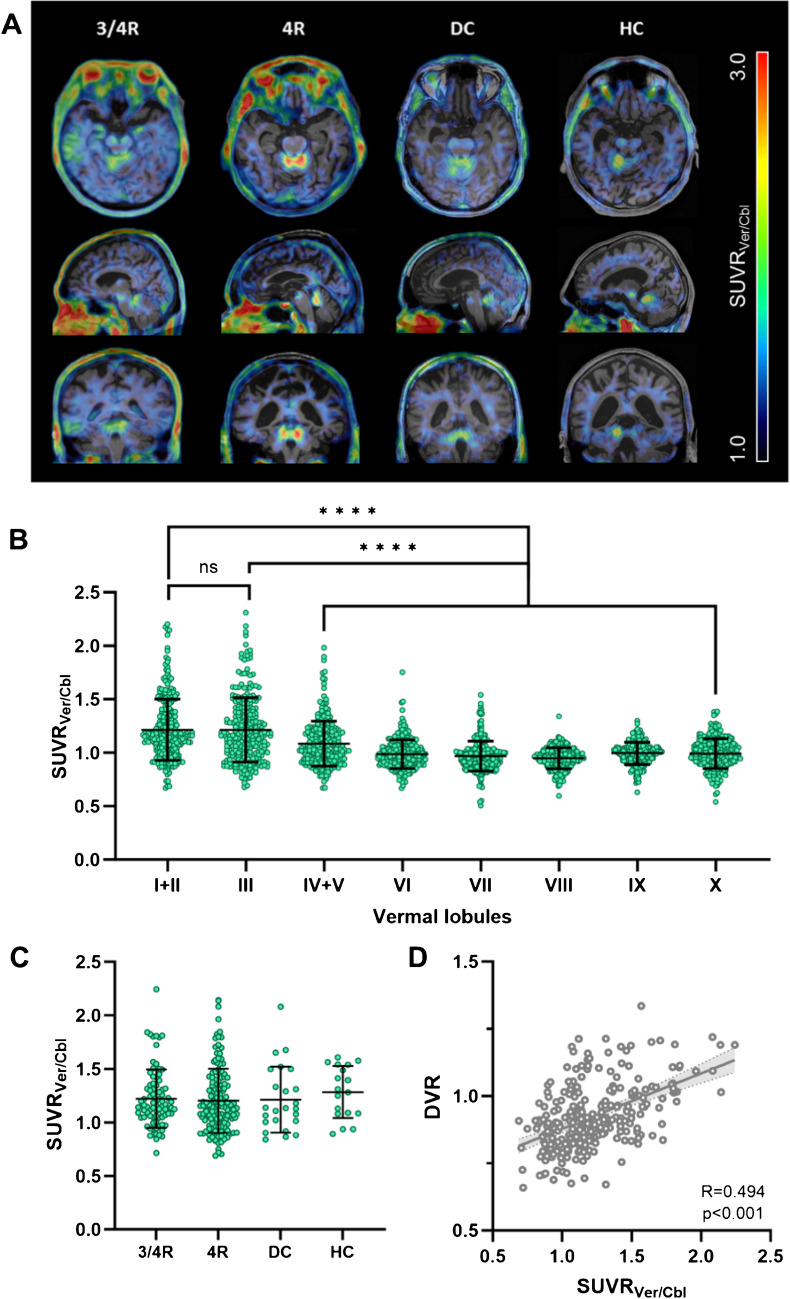
Group-wise comparison of vermal [^18^F]PI-2620 binding. **A** Representative examples of [^18^F]PI-2620 tau-PET scans 30–60 min post injection, with one case per cohort exhibiting elevated vermal tracer uptake. **B** Comparison of [^18^F]PI-2620 binding among the vermal lobules I-X including all participants and using ANOVA. **C** Comparison of vermal [^18^F]PI-2620 binding (lobules I-III) between the four cohorts using ANOVA. **D** Correlation between vermal [^18^F]PI-2620 SUVR_Ver/Cbl_ and DVR

Among all participants in our study (n = 274), we frequently observed a strong [^18^F]PI-2620 PET signal in the vermal lobules I-III, and with some variability, in adjacent cortical regions of vermis, paravermis and anterior cerebellum. For illustration of the vermal [^18^F]PI-2620 PET signal, exemplary PET images for subjects with high vermal tracer uptake are shown in Fig. 1A. Particularly vermal lobules I-III displayed elevated mean SUVR_Ver/Cbl_ compared to the other vermal lobules (Fig. 1B). Throughout all participants, 16.1% (n = 44) exhibited a signal in the vermal lobules I-III with SUVR_Ver/Cbl_ > 1.5.

To further investigate the extent to which vermal tau-PET signals overlap with adjacent brain regions, we conducted a median split (MS) of group DVR values from tau-negative disease and healthy control cohorts. This approach allowed us to assess the influence of vermal tracer uptake independent of actual tau pathology. Each cohort was divided into two subgroups based on vermal tracer uptake, categorized as high (DVR > MS) and low (DVR < MS). We then calculated the %-difference between the average DVR maps of the two subgroups. Visual inspection revealed a notable association of vermal tracer uptake with established target and reference regions implicated in neurodegenerative diseases, including AD [36] and primary tauopathies [4] (Supplemental Fig. 1).

### *Vermal [*^*18*^*F]PI-2620 binding occurs independent from the study group*

For investigation of the relationship between vermal tau-PET signals and specific neurodegenerative diseases, we separated all participants into four cohorts with 3/4R-tauopathies, 4R-tauopathies, tau-negative disease controls and healthy controls. No significant differences were observed among the cohorts comparing SUVR_Ver/Cbl_ (Fig. 1C) and DVR mean values (Supplemental Fig. 2). SUVR_Ver/Cbl_ and DVR correlated strongly with each other (R = 0.494, p < 0.0001, Fig. 1D). Within the 3/4R-tauopathies cohort, no correlations were found between tracer uptake in the vermis and specific Braak regions (Supplemental Fig. 3).

### *[*^*18*^*F]PI-2620 tau-PET reveals sex-dependent but not age-dependent differences of vermal tracer binding*

**Fig. 2:**
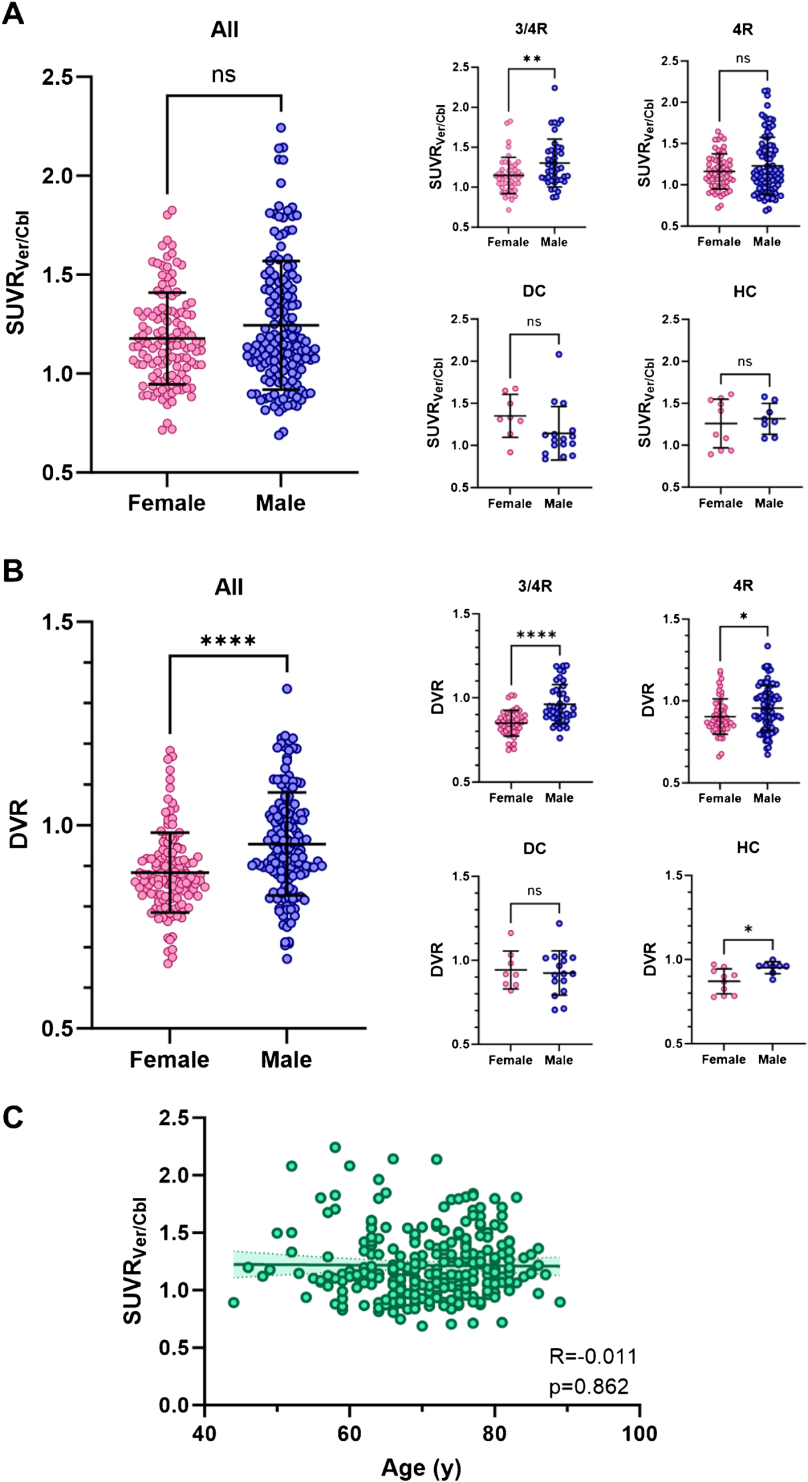
Impact of sex and age on vermal [^18^F]PI-2620 binding. **A** Comparison of vermal [^18^F]PI-2620 SUVR_Ver/Cbl_ (inferior cerebellum reference) between both sexes. **B** Comparison of vermal [^18^F]PI-2620 DVR (inferior cerebellum reference) between both sexes. **C** Correlation between age and vermal [^18^F]PI-2620 binding. R indicates Pearson’s correlation coefficient. Line depicts linear regression with corresponding 95% confidence interval

Among all participants in our study (n = 274), male participants revealed a higher vermal [^18^F]PI-2620 SUVR_Ver/Cbl_ compared to female participants (+ 5.7%, p = 0.058, Supplemental Table 2, Fig. 2A). Between the different cohorts, sex-related difference were most significant in the 3/4R-tauopathies cohort (+ 13.4%, p = 0.009), whereas no significant difference was shown in the 4R-tauopathies (+ 5.7%, p = 0.186), disease control (−15.3%, p = 0.124) and healthy control (+ 4.5%, p = 0.641) cohorts. Similarly, dynamic tau-PET imaging using [^18^F]PI-2620 DVR highlighted significant sex-related differences (+ 7.9%, p < 0.0001), predominantly in the 3/4R-tauopathies cohort (+ 13.3%, p < 0.0001), while the other cohorts exhibit low (4R-tauopathies: 5.7%, p = 0.015; healthy controls: 9.3%, p = 0.012) or no (disease controls: −1.9%, p = 0.744) significant difference between both sexes (Fig. 2B). The observed group differences remained largely consistent even after accounting for age as a covariate in the analysis of vermal sex differences (Supplemental Table 2). Similarly, the differences between male and female participants remained consistent after correcting for the study group, with respect to SUVR_Ver/Cbl_ (p = 0.059 vs. p = 0.058) and DVR (p < 0.0001 vs. p < 0.0001).

Furthermore, we did not observe a significant relationship between [^18^F]PI-2620 SUVR_Ver/Cbl_ and age (R = −0.011, p = 0.862, Fig. 2C), nor with p-Tau in CSF (R = −0.038, p = 0.663, (Supplemental Fig. 4).

### *Vermal [*^*18*^*F]PI-2620 uptake is associated with reduced tracer clearance*

**Fig. 3:**
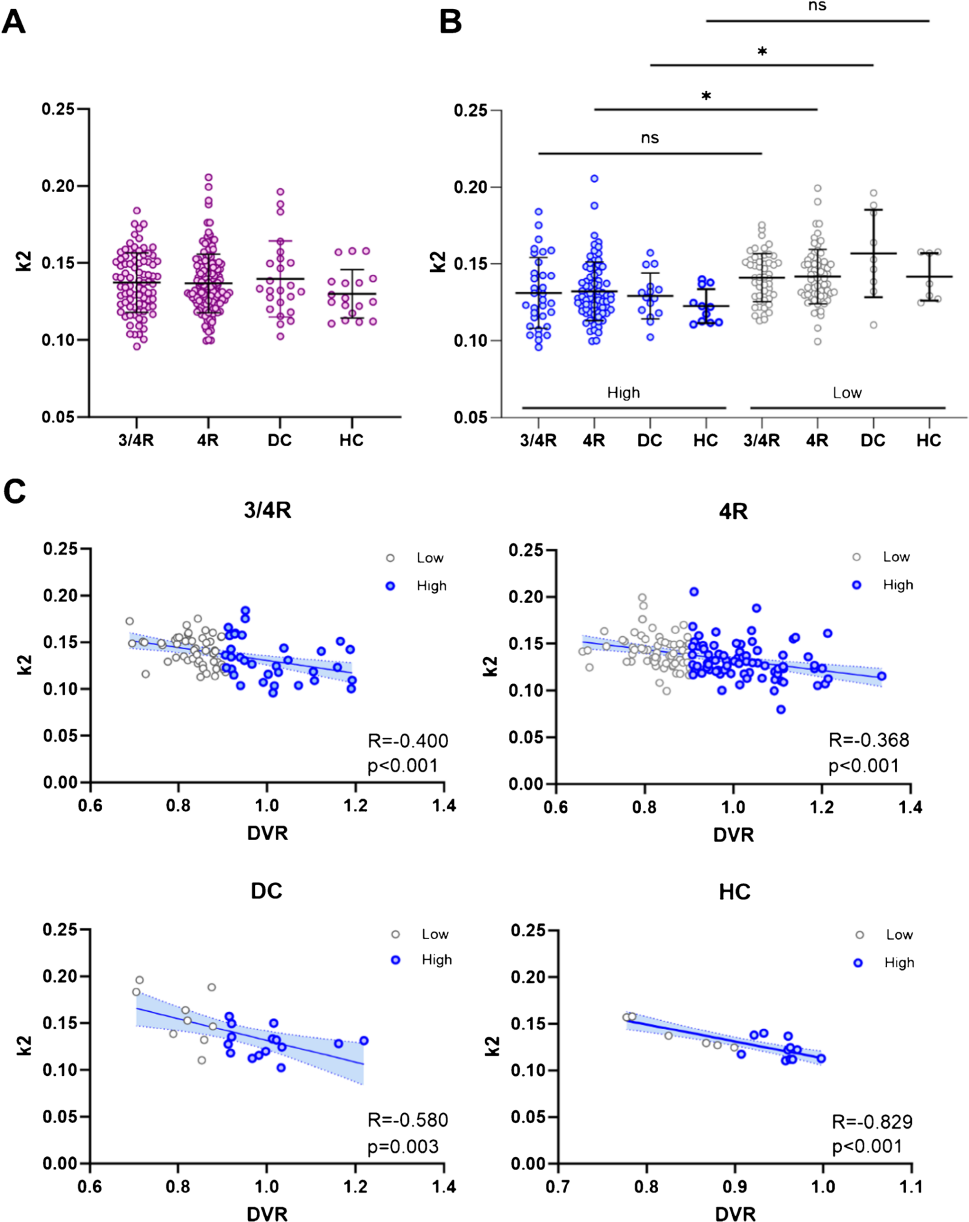
Kinetic properties of vermal [^18^F]PI-2620 PET signal. **A** Comparison of vermal (lobules I-III) tracer efflux (k2) among all four groups. **B** Comparison of vermal (lobules I-III) tracer efflux (k2) between subgroups after median split, categorized into either high (upper 50%) or low (lower 50%) tracer binding. **C** Correlation analyses between vermal tracer uptake and efflux. R indicates Pearson’s correlation coefficient. Lines represent linear regression analyses and shaded areas correspond to 95% confidence intervals

We examined kinetic properties of vermal [^18^F]PI-2620 signal and compared them among the four cohorts. First, tracer clearance (k2), a surrogate for tracer efflux, revealed no significant differences between the cohorts [10] (Fig. 3A). Next, we performed median split (MS) of corresponding group-DVR (threshold = 0.906) and separated each cohort into two distinct subcohorts to compare participants exhibiting high (DVR > MS, n = 137) and low (DVR < MS, n = 137) vermal tracer uptake. Patients with elevated vermal [^18^F]PI-2620 DVR showed significantly lower tracer efflux in the 4R-tauopathies (p = 0.037) and disease control (p = 0.011) cohort. On the contrary, tracer efflux was not significantly altered among the subgroups of 3/4R-tauopathies (p = 0.240) and healthy controls (p = 0.401; Fig. 3B). Strikingly, DVR and k2 of the vermis were significantly associated in all cohorts (3/4R-tauopathies: p = 0.0001; 4R-tauopathies: p < 0.0001; disease controls: p = 0.003; healthy controls: p < 0.0001; Fig. 3C). Furthermore, we investigated the relative perfusion (R1) of [^18^F]PI-2620 as a biomarker of neuronal injury [37]. We found no significant association between vermal tracer uptake and R1 in each cohort, respectively (Supplemental Fig. 5).

### *Vermal [*^*18*^*F]PI-2620 tau-PET signal correlates with abundance of leptomeningeal melanocytes in deceased patients*

We investigated the extent to which the in vivo vermal tau-PET signal reflects the accumulation of tau pathology versus potential contributions from off-target binding to other structures. To this end, we analysed a cohort of eight deceased patients who underwent [^18^F]PI-2620 PET imaging in vivo, with subsequent donation of their brains for autopsy [8]. Six patients were classified as definite PSP, one of whom was found to have an ante-mortem subarachnoid haemorrhage. Two patients were classified as TAR DNA-binding protein 43 (TDP-43)-positive frontotemporal lobar degeneration FTLD-TDP: one FTLD/MND-TDP; one FTLD-TDP related to a TANK-binding kinase 1 (TBK1) mutation. Notably, four individuals with primary PSP also showed AD-related tau co-pathology at varying Braak stages (Supplemental Table 1). Additionally, we included one case with [^18^F]THK-5351 PET, which exhibited elevated vermal tracer signal, for histological assessment (Supplemental Fig. 6).

**Fig. 4:**
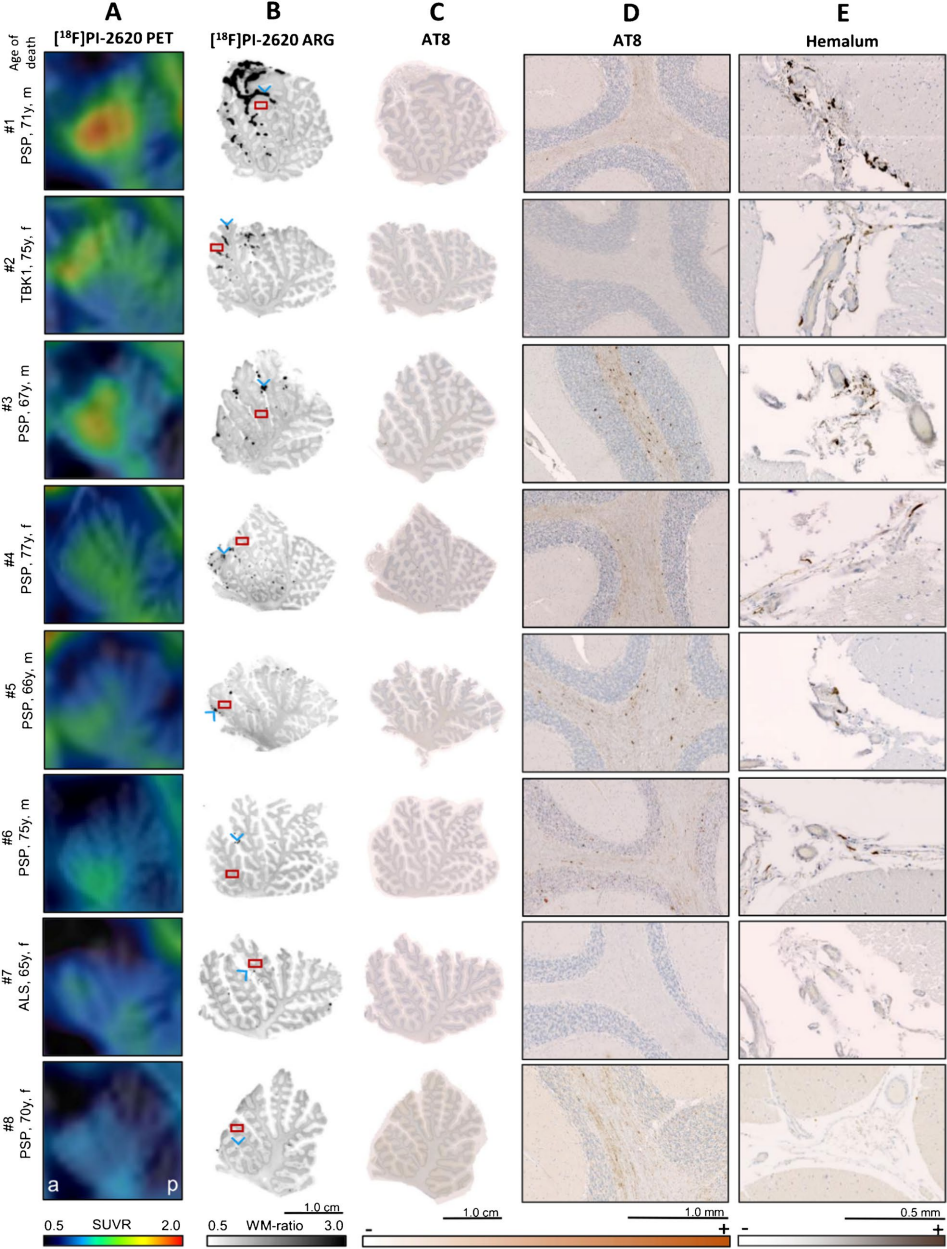
Correlation of in vivo PET signals with autoradiography signals, AT8 immunoreactivity and distribution of melanocytes in the leptomeninges. **A** Sagittal [^18^F]PI-2620 PET images focused on the anterior lobe of the vermis, displayed upon an T1 MRI. Left = anterior; right = posterior. **B** [^18^F]PI-2620 autoradiography of vermal tissue samples. ARG = autoradiography. **C** AT8 immunohistochemistry of adjacent vermal tissue samples to them used in autoradiography. **D** AT8 immunohistochemistry. Zoomed-in image segments correspond to red-boxes visualized in B. **E** Hemalum counterstaining. Representative image segments of regions with visual elevated autoradiography signal, as indicated by blue arrowheads in B

The vermal [^18^F]PI-2620 PET signal, predominantly localized to the lingula (lobules I and II) and central lobule (lobule III) of the vermis, was co-localized with regions of strong autoradiographic signal (Fig. 4A, B). These autoradiographic signal spots were primarily observed within the fissures of the corresponding vermal lobules (Fig. 4B). Hemalum staining revealed dark pigmented-laden cells within the leptomeninges (Fig. 4E). Abundance and distribution of leptomeningeal pigmented cells visually correlated with the intensity and localization of the autoradiographic signal.

**Fig. 5:**
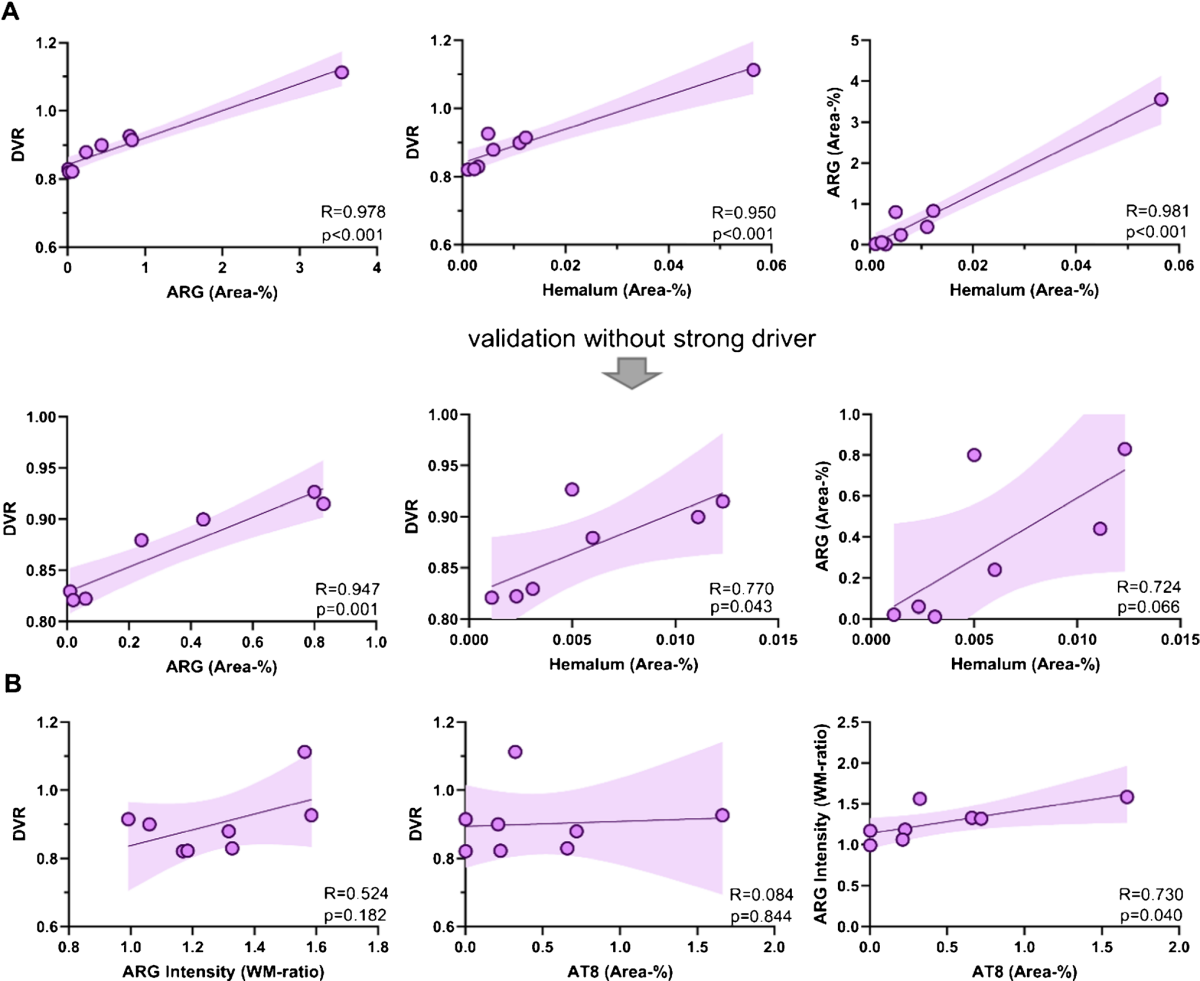
Quantitative correlation between autoradiography, immunohistochemistry and tau-PET DVR in autopsy samples. **A** Relationship between [^18^F]PI-2620 DVR and leptomeningeal melanocytic signal (area-%) in autoradiography and hemalum staining with and without the exclusion of one outlier. Area (%) = area of [^18^F]PI-2620 binding in leptomeninges relative to the area of full size section in autoradiography. **B** Association between tau-related autoradiography signals, tau-PET and AT8 occupancy in vermal white matter subfields. Lines are linear regressions, and shaded areas correspond to 95% confidence intervals. R indicates Pearson’s correlation coefficient

Furthermore, we observed a strong association between autoradiographic [^18^F]PI-2620 binding in the leptomeninges and the in vivo PET signal (DVR: R = 0.978, p < 0.001; SUVR_Ver/Cbl_: R = 0.831, p = 0.011). Across modalities, a strong association was observed between quantitative melanin content in hemalum staining and tau-PET (DVR: R = 0.950, p < 0.001; SUVR_Ver/Cbl_: R = 0.740, p = 0.036) as well as autoradiography (R = 0.981, p < 0.001) (Fig. 5A, Supplemental Fig. 8A). This observation remained largely significant even after excluding one outlier, which exhibited markedly elevated signals in autoradiography, hemalum staining and PET.

In addition, we explored if in vivo signals of [^18^F]PI-2620 are also determined by tau pathology. Therefore, we compared the localisation of PET signals with the distribution of tau deposits in AT8-stained slices. All tauopathy cases showed a range of mild to strong visual and quantitative AT8 occupancy in the vermal white matter of the whole anterior lobe of vermis (Fig. 4C), whereas PET signal primarily occurred in the anterior portion of vermis (lobules I-III, Fig. 4A) even in one non-tauopathy case (#2). Quantitative analysis revealed no significant correlation between tau-related autoradiographic signal and [^18^F]PI-2620 PET signal (DVR: R = 0.524, p = 0.182; SUVR_Ver/Cbl_: R = 0.347, p = 0.400). Similarly, no significant relationship was observed between AT8 occupancy and tau-PET (DVR: R = 0.084, p = 0.844; SUVR_Ver/Cbl_: R = 0.076, p = 0.858). However, AT8 occupancy showed a significant correlation with autoradiography ratios (R = 0.730, p = 0.040) (Fig. 5B, Supplemental Fig. 8B), speaking for a low increase of [^18^F]PI-2620 signals in presence of AT8-positive oligodendrocytes in the cerebellar white matter which does not translate into significant in vivo binding. To substantiate this hypothesis, we performed a regression analysis with AT8 and hemalum staining as predictors for tau and melanocytic binding and found that only leptomeningeal melanocytes (β = 0.984, p < 0.0001) but not tau (β = 0.231, p = 0.064) significantly explained the vermal [^18^F]PI-2620 signal (F = 51.41, p < 0.0001).

In one autopsy case with ante-mortem subarachnoid haemorrhage, we detected iron deposits within the leptomeninges. Visual comparison of Prussian blue staining and [^18^F]PI-2620 autoradiography on adjacent tissue slices revealed a strong association between tracer binding in areas containing melanocytes but no relevant tracer binding in areas of iron deposits (Supplemental Fig. 9).

## Discussion

This study represents the first comprehensive identification of the origin of the frequent [^18^F]PI-2620 PET signals within the vermal lobules I-III and adjacent cortical regions, including the vermis, paravermis, and anterior cerebellum. Our primary objective was to assess the contribution of tau deposits to the vermal [^18^F]PI-2620 uptake and to investigate potential off-target structures. As a major achievement, our findings, derived from tau-PET imaging data and histopathological analyses, identify melanocytes in the leptomeninges overlying the anterior vermis as the principal source of the vermal [^18^F]PI-2620 signal. In contrast, tau accumulation and iron deposits appear to contribute only very limited to vermal [^18^F]PI-2620 binding.

Several previous studies have reported on an elevated PET signal in the vermis, the anterior and superior cerebellum, occurring across different tau tracers of the first and second generation. These include [^18^F]AV-1451 [22], [^18^F]JNJ-067 [23], [^18^F]RO-948 [24, 26], [^18^F]MK-6240 [25, 26] and [^18^F]PI-2620 [27]. To investigate the origin of the vermal tau-PET signal, we conducted an extensive set of analyses, including static and dynamic tau-PET imaging using the second generation radioligand [^18^F]PI-2620, along with examination of tissue samples from deceased patients with neurodegenerative diseases.

Initially, we assessed the ability of [^18^F]PI-2620 PET to measure and localize tracer uptake in the vermal and adjacent regions. Our primary finding revealed elevated [^18^F]PI-2620 PET signals, particularly in vermal lobules I-III, with some variability extending to adjacent regions of the vermis, paravermis, and anterior cerebellum. Notably, vermal lobules I-III demonstrated higher mean SUVR_Ver/Cbl_ compared to other vermal lobules, suggesting preferential tracer accumulation in these areas. The selection of appropriate reference regions is critical to minimize spill-in effects and subsequent accurate interpretation of tau-PET signal sources. While earlier studies utilized the entire cerebellar grey matter as a reference region [38], more recent approaches have excluded the superior cerebellum, including the vermis, to mitigate these risks [4]. Also, tau-accumulating structures, such as the dentate nucleus, are commonly excluded. Emerging data-driven methodologies have identified alternative reference regions, such as the fusiform gyrus and cerebellar crus, which may provide enhanced specificity [39]. We examined the impact of vermal off-target signals by a regional comparison of assumed tau-negative individuals with high and low vermal [^18^F]PI-2620 uptake. These analyses revealed potential spill-in effects from off-target binding in the vermal region to adjacent target regions associated with AD (e.g., occipital and temporal lobes) and PSP (e.g., dorsal midbrain, dentate nucleus) [4, 36] in individuals with strong vermal tau tracer uptake. As a consequence, vermal tau-PET uptake should be carefully considered regardless of clinical diagnosis or the presence of tau pathology, and warrants the use of eroded masks to exclude spill-in effects into relevant target regions depending on the studied cohorts.

Furthermore, a highly significant association was observed between static (SUVR) and dynamic (DVR) tau-PET imaging within these target regions of the vermis. Consistent with the lack of correlation between tracer delivery (R1) and SUVR_Ver/Cbl_ uptake, perfusion-related alterations as a source of off-target tracer uptake were ruled out.

Next, we assessed the impact of patients’ demographics on vermal tau-PET signal. There was no association between SUVR_Ver/Cbl_ and age, suggesting that the target structure for [^18^F]PI-2620 does not accumulate significantly upon aging, which is in contrast with expected age-related increase of tau burden [40, 41]. Sole investigation of the AD cohort revealed no association between vermal and cortical tracer uptake in the individual Braak regions, indicating that the vermal tracer signal does not correspond to progressive tau pathology. Moreover, elevated levels of p-Tau did not correlate with high SUVR_Ver/Cbl_ across all cohorts, supporting the hypothesis that the vermal tau-PET signal is primarily influenced by off-target binding. This observation aligns with the established increase in p-Tau associated with the progression of tau pathology in the brain [42, 43]. Additionally, [^18^F]PI-2620 demonstrated comparable accumulation in the vermal regions of tau-negative and tau-positive individuals. In the latter, SUVR_Ver/Cbl_ did not show a significant difference between the 3/4R-tauopathies and 4R-tauopathies, despite previous studies strongly indicating that [^18^F]PI-2620 could differentiate between these two disease entities [10, 11].

The evaluation of the kinetic properties of [^18^F]PI-2620 reinforced our hypothesis of a signal primarily driven by off-target binding, as evidenced by the similar binding affinity and tissue clearance (k2) observed across the different cohorts. Consequently, we examined whether higher binding affinity correlates with the abundance of tau within these cohorts as significant [^18^F]PI-2620 binding to tau deposits, particularly to 3/4R-tau, is characterized by reduced tissue clearance and correspondingly lower k2 values, as previously reported [10]. For this purpose, we conducted a median split, dividing participants within each cohort into two subgroups based on high or low vermal tau-PET signal. Notably, the comparison between high and low vermal signal subgroups within each cohort demonstrated only weak or non-significant differences in tracer efflux. This finding suggests a low level of tau-related [^18^F]PI-2620 accumulation, even within both tauopathy cohorts.

As a central component of our study, histological experiments identified melanin-containing cells within the vermal leptomeninges as the primary binding target for [^18^F]PI-2620. Hemalum-stained slices revealed these pigment-laden cells in the leptomeninges overlying the anterior lobe of the vermis and within its fissures, primarily adjacent to the lingula and the central lobule, with a lesser presence near the culmen. The distribution and abundance of these cells visually and quantitatively correlated significantly with the location and intensity of both [^18^F]PI-2620 signal spots observed in autoradiography and [^18^F]PI-2620 uptake in tau-PET scans. Contrarily, tau-related uptake, as measured by AT8 occupancy, did not contribute significantly to the vermal tau-PET signal, supporting the conclusion that the observed visual signal is predominantly driven by melanocytes in the leptomeninges. This finding is consistent with observations from our CBS autopsy case, which exhibited in vivo vermal [^18^F]THK-5351 PET signal. As previously reported, both tau tracers included in our study have not only demonstrated recurrent tracer uptake in the superior cerebellum and vermis, but are also known to bind to subpial melanin-containing structures and neuromelanin in the substantia nigra [9, 17, 20, 24, 44] (for [^18^F]JNJ-067, binding to melanin has not yet been verified). Additionally, another recent study has reported on [^18^F]MK-6240 binding to leptomeningeal melanocytes overlying the cerebellum [20]. Therefore, binding to melanin and neuromelanin is a well-known lack of tau tracer specificity and has already been observed in leptomeningeal structures. Although histopathological studies detected leptomeningeal pigment cells over the ventrolateral surfaces of the medulla oblongata and to a small extent overlying the cerebellum and cerebral hemispheres [45], reports on increased density of these cells in the vermal region and superior cerebellum are currently absent in the literature and should be examined more closely given the frequent accumulation of tau radioligands in these regions. This is particularly important due to the impact of elevated signal in the vermis and adjacent cerebellum on the accuracy of tau-PET quantification when using the cerebellum as a reference region [22, 46].

As an interesting discovery, we detected sex differences in vermal [^18^F]PI-2620 binding, revealing higher tracer uptake in men compared to women across all subjects. Sex-related differences were not consistently observed across the individual cohorts. In the evaluation of SUVR_Ver/Cbl_, significant sex differences were only shown in the 3/4R-tauopathies cohort. However, the analysis of DVR revealed enhanced sex-related differences that were quantitatively measurable in both the 4R-tauopathies and healthy control groups, with the most pronounced effects still evident in the 3/4R-tauopathies cohort. Noteworthy, this finding may have been distorted due to the relatively small size of the 3/4R-tauopathies cohort. Nevertheless, male participants generally exhibited a greater prevalence of high extremes in vermal [^18^F]PI-2620 signal. A prior study involving cognitively unimpaired, amyloid-β negative participants identified increased off-target binding in men compared to women in the upper regions of the cerebellum using [^18^F]RO-948 and [^18^F]MK-6240 [26]. Notably, this study also revealed a regional inconsistency between the tracers, showing elevated tau-PET uptake in the skull and meninges of female participants.

The inhomogeneity of tracer signal in the vermis and superior cerebellum has also been recognized in our study and may be attributed to the variability in the presence and distribution of melanin-containing cells among individuals. However, the underlying reasons for elevated vermal signal in men, most pronounced in AD, remain unclear. Our analysis of tracer kinetics indicates that tau deposits are likely less responsible for vermal uptake, consistent with the aforementioned study that confirmed sex-related off-target binding findings in participants without cognitive disorders [26].

It is conceivable, though not yet verified in the literature, that sex hormones may influence leptomeningeal melanogenesis. Another potential factor could be iron deposits resulting from subarachnoid bleeding, as observed in one autopsy sample from our study, which correlated with a visually weak binding of [^18^F]PI-2620 in autoradiography. Noteworthy, the anterior vermis is known to be a predilection site for siderotic depositions [47]. Although men tend to have higher iron levels, the potentially higher iron content in haemorrhagic deposits with an effect on sex-related tracer accumulation remains speculative. An autoradiographic study on flortaucipir found no detectable binding to hemosiderin deposits in a superficial siderosis case suggesting that the age of the haemorrhages may lead to varying extents of tracer binding [19].

There is evidence that iron homeostasis is linked to melanogenesis. Iron overload is associated with increased skin pigmentation due to increased melanin levels [48] and a previous study reported on the ability of iron to upregulate melanogenesis [49]. In addition, melanin is attributed a role in protecting tissues from harmful factors, including metal ions [21, 50]. Concerning the sex-related differences observed primarily in the AD cohort, there may be a relationship between bleeding of leptomeningeal vessels and cerebral amyloid angiopathy (CAA), a condition closely associated with AD [51]. Reports indicate that male patients affected by CAA exhibit a higher local iron burden compared to their female counterparts [52].

Even though the high number of participants strengthened our findings, there are some limitations to consider. A primary limitation of our autopsy study is the absence of tissue samples from patients with clinically diagnosed AD, which would have provided valuable insights into the sex-related differences in vermal off-target binding. Additionally, this study is cross-sectional in nature and does not allow for an investigation of individual vermal signal progression, despite our finding that there was no correlation between vermal [^18^F]PI-2620 PET signal and age. Moreover, we acknowledge the relatively low number of total autopsy cases as well as healthy controls with tau-PET imaging, which may limit the generalizability of our findings.

## Conclusion

To conclude, we identified melanocytes in the leptomeninges overlying the anterior vermis and cerebellum as the primary target for vermal [^18^F]PI-2620 PET binding. In contrast, tau aggregates and iron deposits contributed only minimally to the observed signal. The association between male sex and elevated vermal tau-PET signals across different radioligands may be linked to sex hormones or iron deposits; however, this remains speculative and warrants further investigation. Importantly, our findings highlight the importance of excluding the vermal region from tau-PET analyses due to specific off-target binding.

## Supplementary Information

Below is the link to the electronic supplementary material.

ESM 1 (DOCX 8.80 MB)

ESM 2 (DOCX 27.0 KB)

## Data Availability

The datasets generated during and/or analyzed during the current study are available from the corresponding author on reasonable request. CP, JL, JH, GH, NF, and MB were funded by the Deutsche Forschungsgemeinschaft (DFG) under Germany’s Excellence Strategy within the framework of the Munich Cluster for Systems Neurology (EXC 2145 SyNergy, ID 390857198).
