# Supplementary material for: Exploring the origins of frequent tau-PET signal in vermal and adjacent regions
Source: Eur J Nucl Med Mol Imaging. 2025 Mar 18;52(10):3519–33. doi: 10.1007/s00259-025-07199-x (PMC12316827; doi:10.1007/s00259-025-07199-x)
Supplement: Supplementary file 1 — (DOCX 8.80 MB) [file 259_2025_7199_MOESM1_ESM.docx]

**Supplemental Material to:**

**Exploring the origins of frequent tau-PET signal in vermal and adjacent regions**

Agnes Kling^1^, Julia Kusche-Palenga^1^, Carla Palleis^2,3,4^, Alexander Jäck^2,3^, Alexander M Bernhardt^2,3^, Lukas Frontzkowski^1,5^, Sebastian N Roemer^3,5^, Luna Slemann^1^, Mirlind Zaganjori^1^, Maximilian Scheifele^1^, Lars Paeger^2^, Gérard N Bischof^7,8^, Thilo van Eimeren^7,8,9,10^, Alexander Drzezga^7,8,10^, Osama Sabri^11^, Michael Rullmann^11^, Henryk Barthel^11^, Johannes Levin^2,3,4^, Jochen Herms^2,4,6^, Nicolai Franzmeier^4,5,12^, Günter Höglinger^2,3,4^, Sigrun Roeber^6^, Matthias Brendel^1,2,4^, and Johannes Gnörich^1,2^

^1^Department of Nuclear Medicine, LMU Hospital, Ludwig Maximilian University of Munich, Munich, Germany
^2^German Center for Neurodegenerative Diseases (DZNE) Munich, Munich, Germany
^3^Department of Neurology, LMU Hospital, Ludwig-Maximilians-University of Munich, Munich, Germany
^4^Munich Cluster for Systems Neurology (SyNergy), Munich, Germany

^5^Institute for Stroke and Dementia Research, LMU University Hospital, LMU Munich, Munich, Germany ^6^Center of Neuropathology and Prion Research, University of Munich, Munich, Germany
^7^Department of Nuclear Medicine**,** University Hospital Cologne, Cologne, Germany
^8^Institute of Neuroscience and Medicine (INM-2), Research Center Jülich, Jülich, Germany
^9^Department of Neurology, University Hospital Cologne, Cologne, Germany

^10^German Center for Neurodegenerative Diseases (DZNE), Bonn-Cologne, Germany
^11^Department of Nuclear Medicine, University Hospital Leipzig, Leipzig, Germany
^12^University of Gothenburg, The Sahlgrenska Academy, Institute of Neuroscience and Physiology, Department of Psychiatry and Neurochemistry, Mölndal and Gothenburg, Sweden

**Corresponding author**

Dr. Johannes Gnörich; Marchioninistraße 15, 81377 Munich, +4989440074655, Johannes.Gnoerich@med.uni-muenchen.de

**Supplemental Methods**

*Histochemistry*  Immunohistochemistry was performed on 4 µm thick sections of formalin-fixed and paraffin-embedded tissue samples using standard techniques. The immunohistochemical tau-staining with mouse monoclonal AT8 antibody raised against hyperphosphorylated tau (Ser202/Thr205, 1:200, Invitrogen/Thermofisher, Carlsbad, CA, USA) and the hemalum counterstaining according to manufacturing guidelines (Hoffmann-LaRoche AG, Basel, Switzerland) were carried out semi-automatically on a BenchMark device (Ventana, now Hoffmann-LaRoche, Basel, Switzerland) on adjacent sections of those used in the autoradiography. In one case with ante-mortem subarachnoid haemorrhage, Prussian blue staining was conducted to evaluate iron deposits. Stained sections were digitized at 20x magnification with a Mirax Midi scanner (Zeiss, Carl Zeiss MicroImaging GmbH, Jena, Germany) and evaluated using ZEN 3.4 blue edition software (Zeiss, Jena, Germany). They were used for correlation with vermal [^18^F]PI-2620 autoradiographic signal and PET signal (**Figure 5, Supplemental Figure 8**).

*Autoradiography* For direct comparison with histochemistry, [^18^F]PI-2620 autoradiography from formalin-fixed and paraffin-embedded tissue blocks from nine cases with different neurodegenerative diseases was processed (**Supplemental Table 1**). The anterior lobe of vermis containing lobules I-V was chosen as tissue sample, respectively. For each patient, autoradiography was performed on ≥2 adjacent sections as described previously [1]. In short, deparaffinized sections were incubated for 45 min (21.7 µCi/ml after dilution to a volume of 50 ml with phosphate buffered saline solution, pH 7.4, specific activity 480 ± 90 GBq/µmol). Washing was performed by 30% ethanol/PBS for 1 min, 70% ethanol/PBS for 2 min and PBS for 1 min to remove unbound radiotracer. After drying at room temperature for 60 min, the sections were placed on Fujifilm BAS cassette2 2025 imaging plates. The plates were exposed for approximately 12 h and then scanned at 25.0 µm resolution with the Elysia-raytest equipment (CR-35 BIO, Dürr Medical, Bietigheim-Bissingen, Germany). Resulting images were analysed with a dedicated software (AIDA image analysis, V4.50, Elysia-raytest, Straubenhardt, Germany). To investigate tau-related vermal [^18^F]PI-2620 signal, regions of interest (n=3) were drawn on each sample using the AT8 staining of the adjacent section, thus serving for anatomical definition of subfields in the vermal white matter. An AT8-negative region in the white matter was determined as a reference region. Ratios between subfield target regions and the reference region were calculated and correlated with SUVR_Ver/Cbl_ and vermal DVR extracted from tau-PET data. [^18^F]PI-2620 binding to melanin was quantified by measuring the area of strong signal foci within the leptomeninges in the vermal fissures. The required intensity threshold for each sample was defined by extracting the intensity of an AT8-negative reference region in the white matter. By multiplying this reference value by three and using the resulting value as the intensity threshold, we ensured that only the areas of signal spots in the leptomeninges were included (**Supplemental Figure 7**). Ratios between the area of the leptomeningeal signal and the total tissue area were calculated and correlated with SUVR_Ver/Cbl_ and vermal DVR extracted from tau-PET data. Autoradiographic signal attributed to iron deposits was visually aligned (**Supplemental Figure 9**). An overview of tissue samples from autopsy cases with previous in vivo [^18^F]PI-2620 PET scan (n=8) is shown in **Figure 4**. Additionally, tissue samples from one autopsy case with previous [^18^F]THK-5351 PET are shown in **Supplemental Figure 6**.

**Supplemental Figure 1**


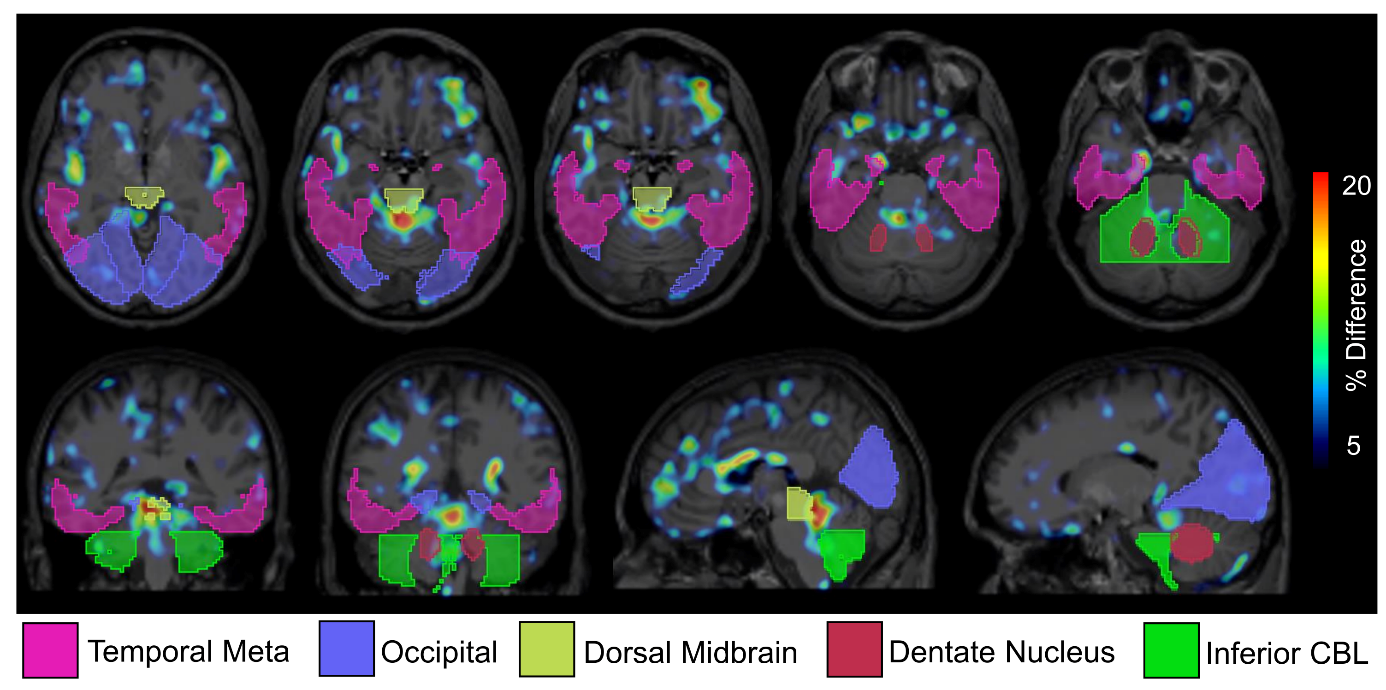
 **Supplemental Figure 1. Vermal tau-PET uptake in relation to adjacent regions of interest.** Axial, coronal, and sagittal slices show % difference maps of tau-negative participants, separated by median split, overlaid on a representative T1 MRI template. Colored regions correspond to distinct target regions of interest near the vermis [2, 3]. Abbreviations: CBL=Cerebellum.

**Supplemental Figure 2**

**
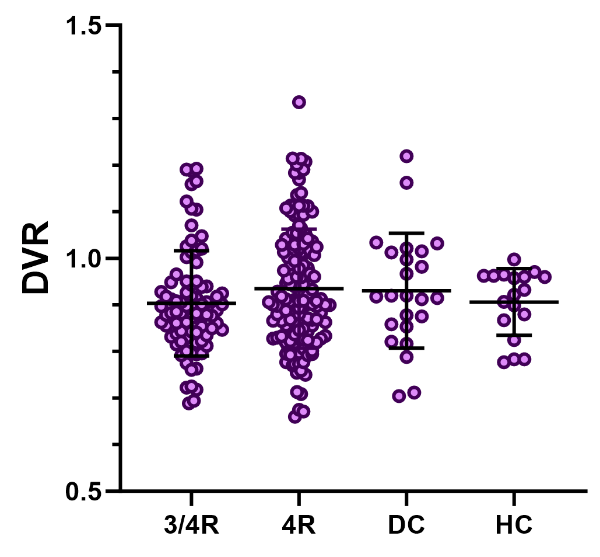
**

**Supplemental Figure 2.** **Comparison of vermal [^18^F]PI-2620 DVR between the four cohorts.**

**Supplemental Figure 3**


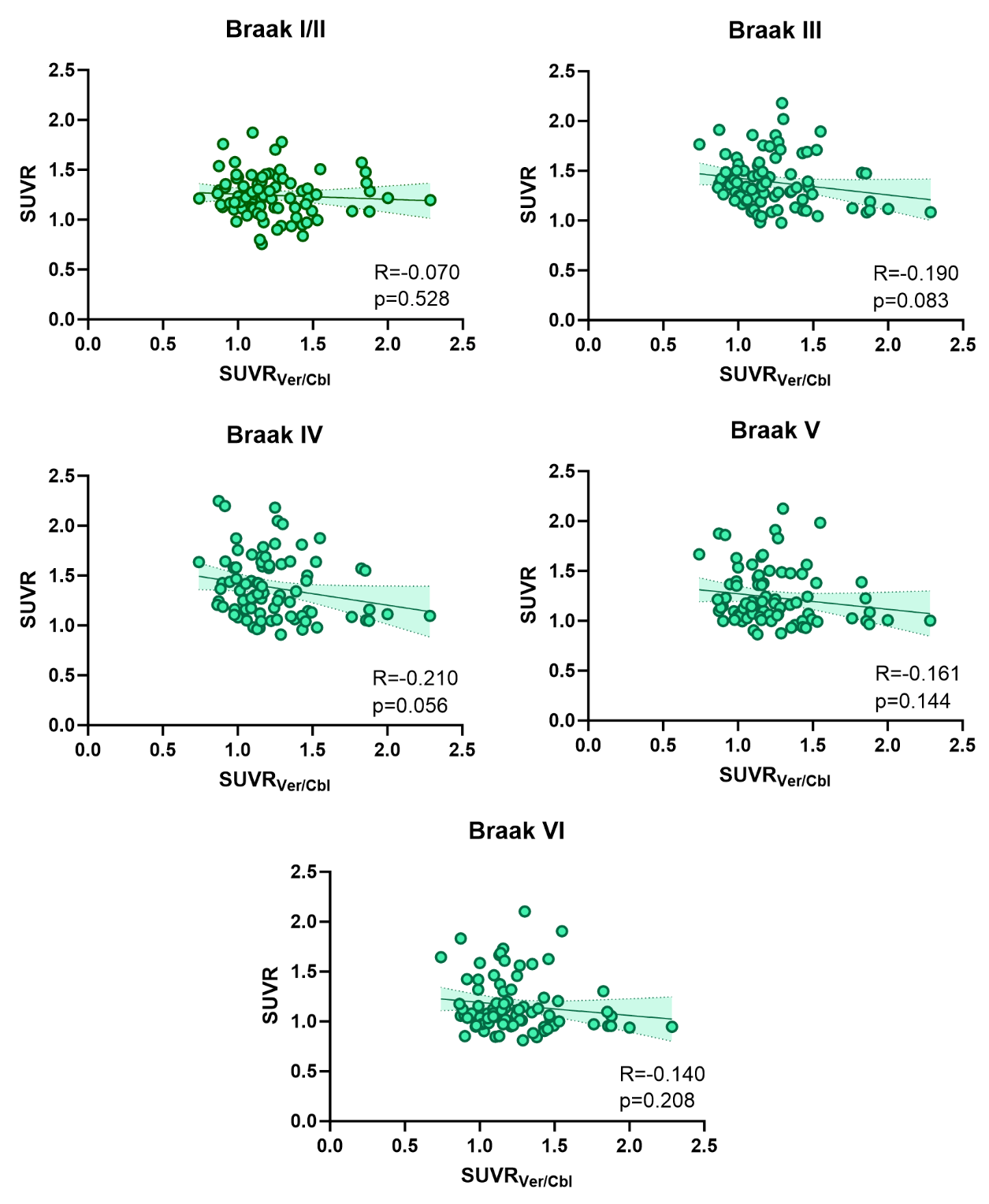


**Supplemental Figure 3. Correlation analyses of [^18^F]PI-2620 uptake between the vermal region and individual Braak regions.** R indicates Pearson’s correlation coefficient. Lines represent linear regression analyses and shaded areas correspond to 95% confidence intervals.

**Supplemental Figure 4**

**
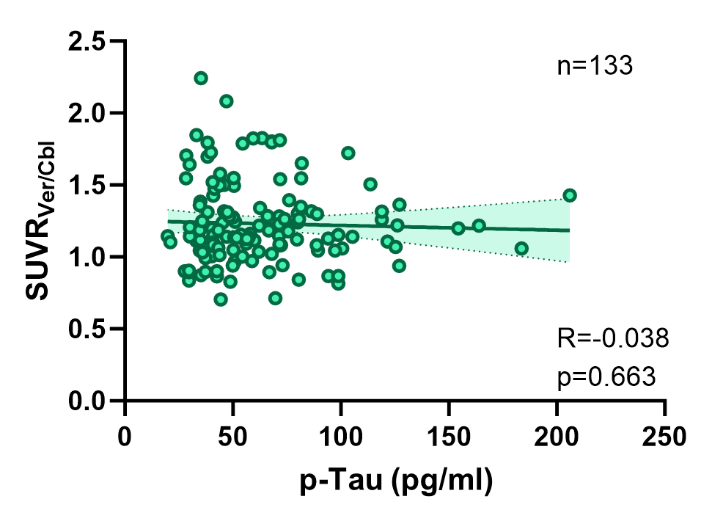
**

**Supplemental Figure 4. Correlation between age and p-Tau_181_ in CSF.** R indicates Pearson’s correlation coefficient across all individuals with available CSF. Line represents linear regression analyses and shaded areas correspond to 95% confidence intervals.

**Supplemental Figure 5**

**
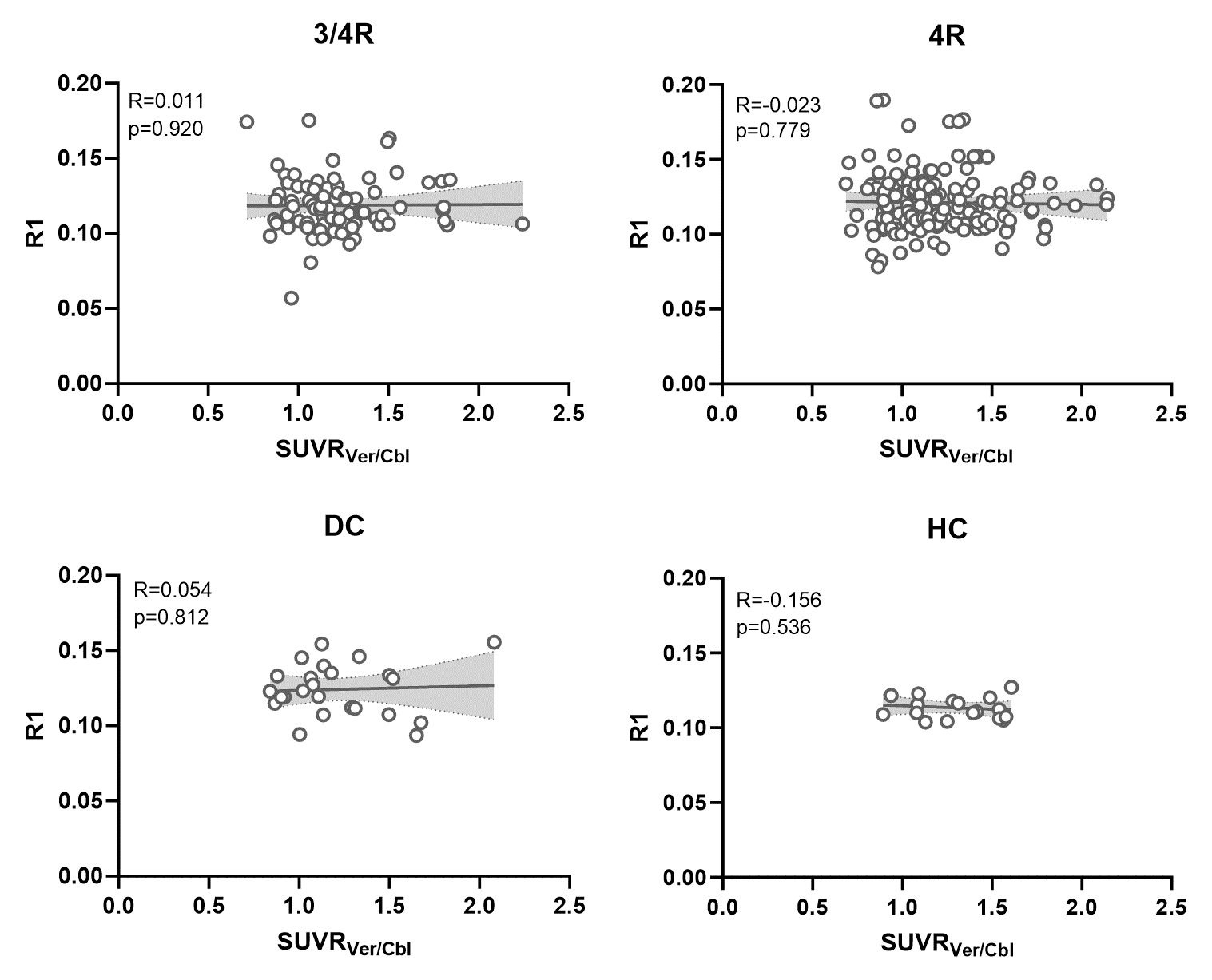
**

**Supplemental Figure 5. Correlation analysis between vermal [^18^F]PI-2620 binding and tracer delivery (R1).** R indicates Pearson’s correlation coefficient. Lines represent linear regression analyses and shaded areas correspond to 95% confidence intervals.

**Supplemental Figure 6**

**
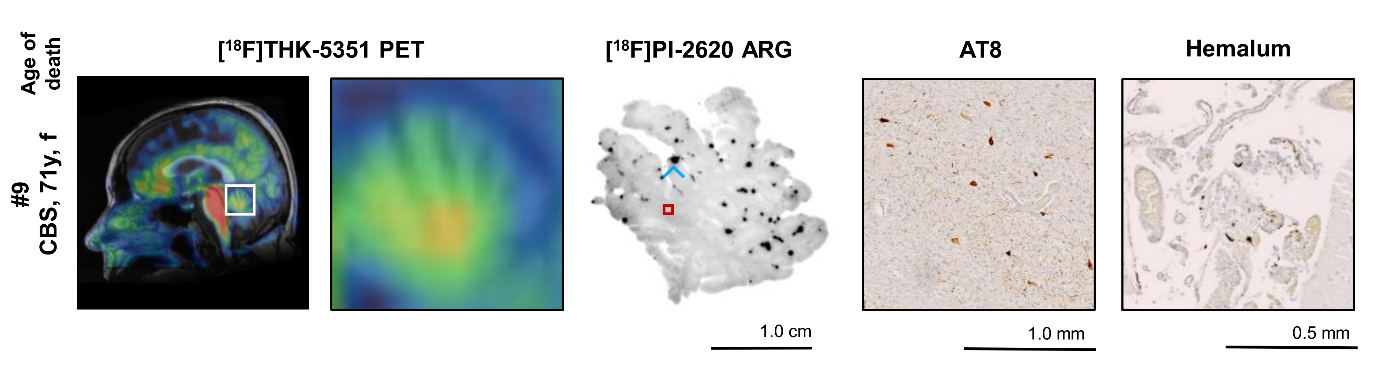
Supplemental Figure 6. Comparison of [^18^F]THK-5351 PET,** **[^18^F]PI-2620 autoradiography and immunohistochemical AT8 and hemalum stainings in one case with CBS.** Sagittal [^18^F]PI-2620 PET image overlaid on a T1-weighted MRI, with an image section zoomed in on the anterior lobe of vermis. The zoomed-in AT8-stained image corresponds to the red-boxed region displayed in the autoradiography (ARG) overview, while the zoomed-in hemalum-counterstained image corresponds to the autoradiography section indicated by the blue arrowhead.

**Supplemental Figure 7**


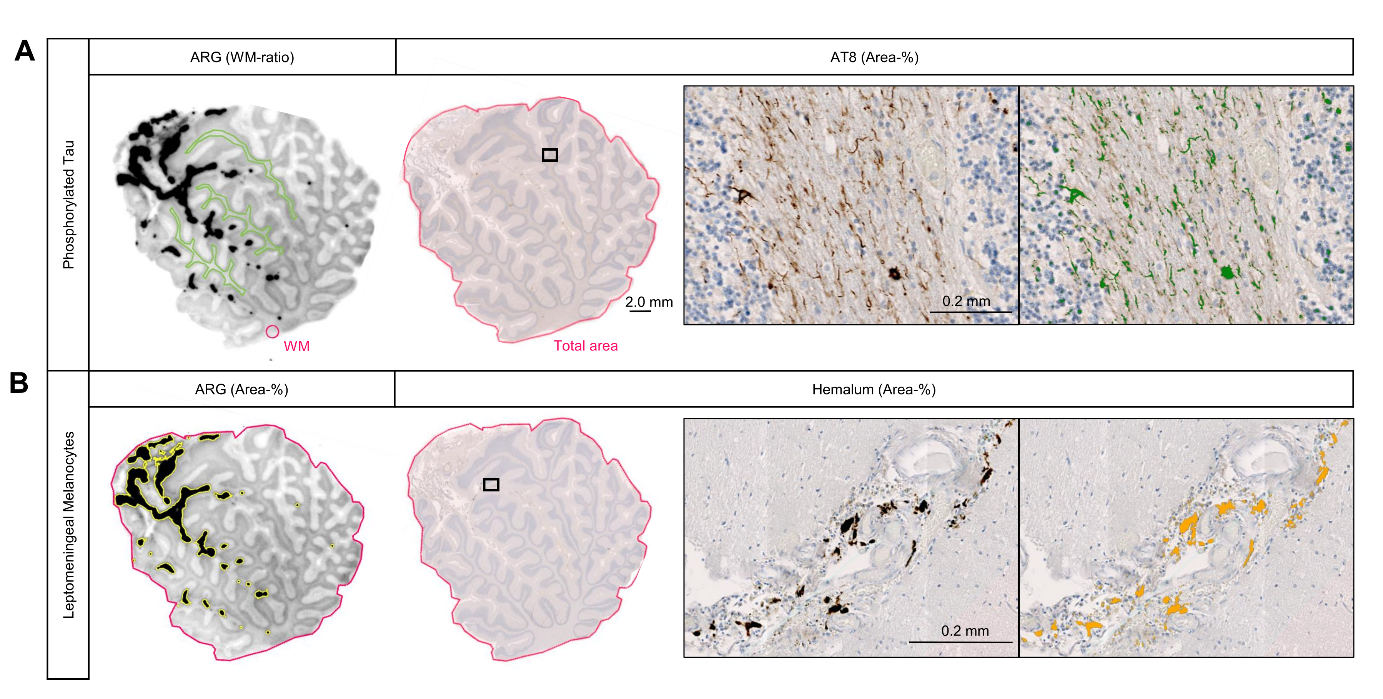


**Supplemental Figure 7. Methodological details of autoradiography, AT8, and hemalum immunohistochemical segmentation.** (**A-B**) Representative images of autopsy case #1 show vermal autoradiography (left), the corresponding AT8- and hemalum-stained sections (middle panel), and detailed high-magnification views with overlays (right panel). (**A**) The upper left panel shows the vermal white matter (light green) in relation to the circular white matter reference region (WM, pink), used for determining autoradiography target-to-white matter ratios. The upper right high-magnification image displays AT8-positive oligodendrocytes (dark green) in the vermal white matter relative to the total vermal area (middle panel, pink). (**B**) The lower left panel depicts leptomeningeal autoradiographic signal (light-yellow) in relation to the total vermal area (pink), while the lower right high-magnification image highlights hemalum-stained leptomeningeal melanocytes (dark yellow) in relation to the total area (middle panel, pink). Consistent masking and automated detection were implemented across all samples using ZEN 3.4 Blue Edition software (Zeiss, Jena, Germany) to ensure precise and reproducible quantification.

**Supplemental Figure 8**

**
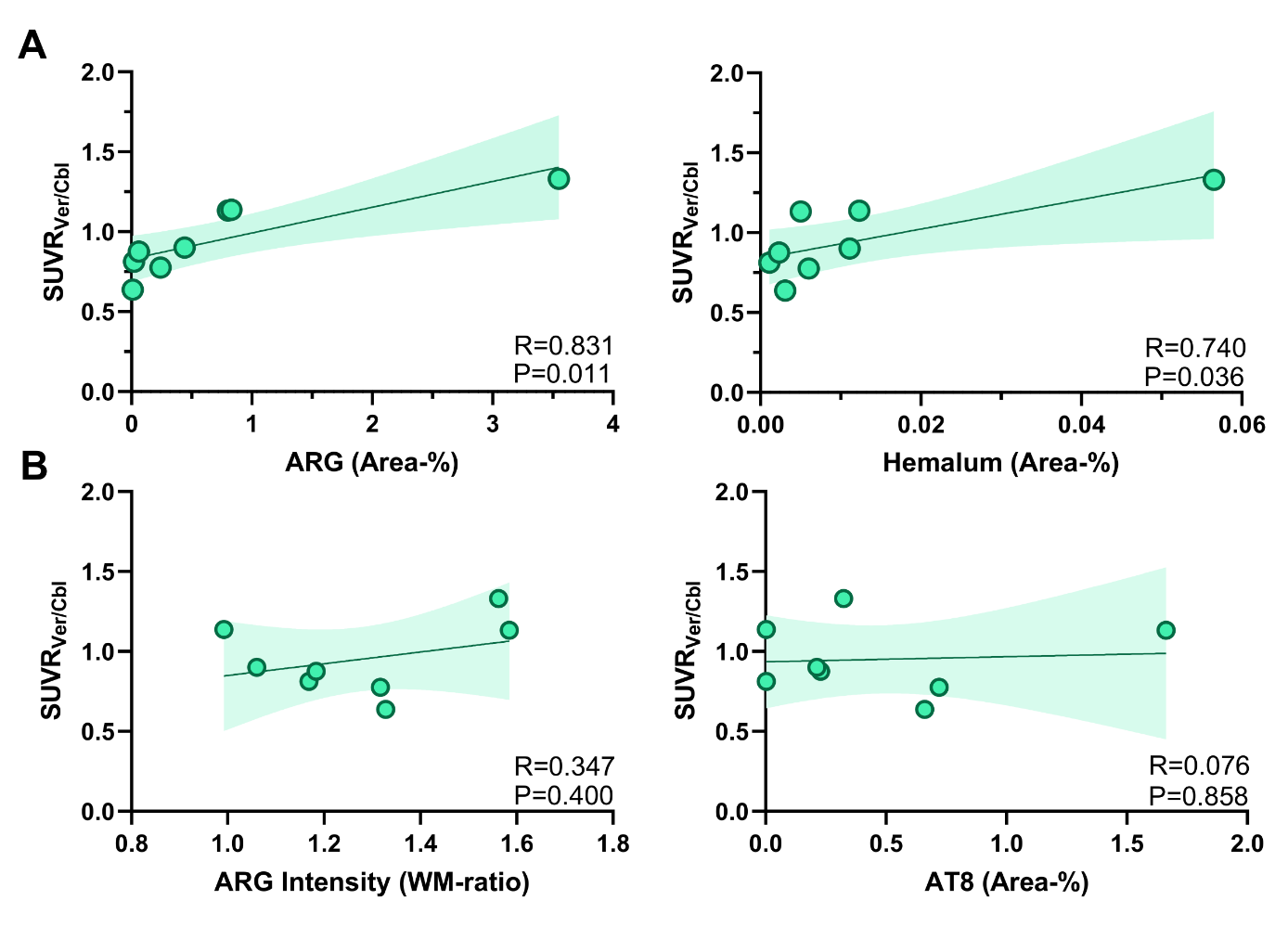
**

**Supplemental Figure 8. Quantitative correlation between autoradiography, immunohistochemistry and tau-PET SUVR in autopsy samples**. R indicates Pearson’s correlation coefficient. Lines represent linear regression analyses and shaded areas correspond to 95% confidence intervals.

**Supplemental Figure 9**


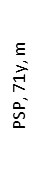


0.1 mm


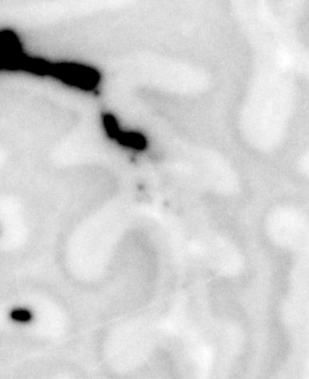


**Prussian blue**


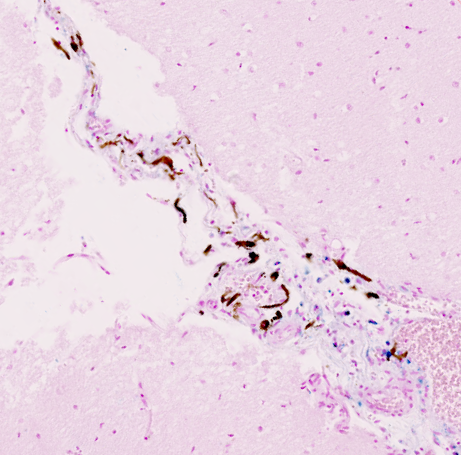

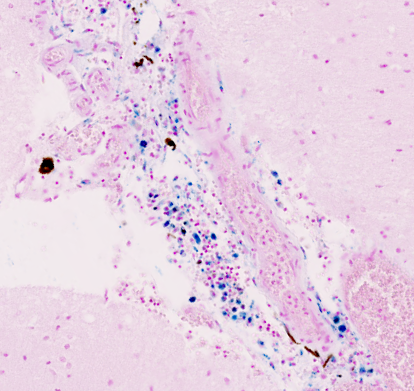

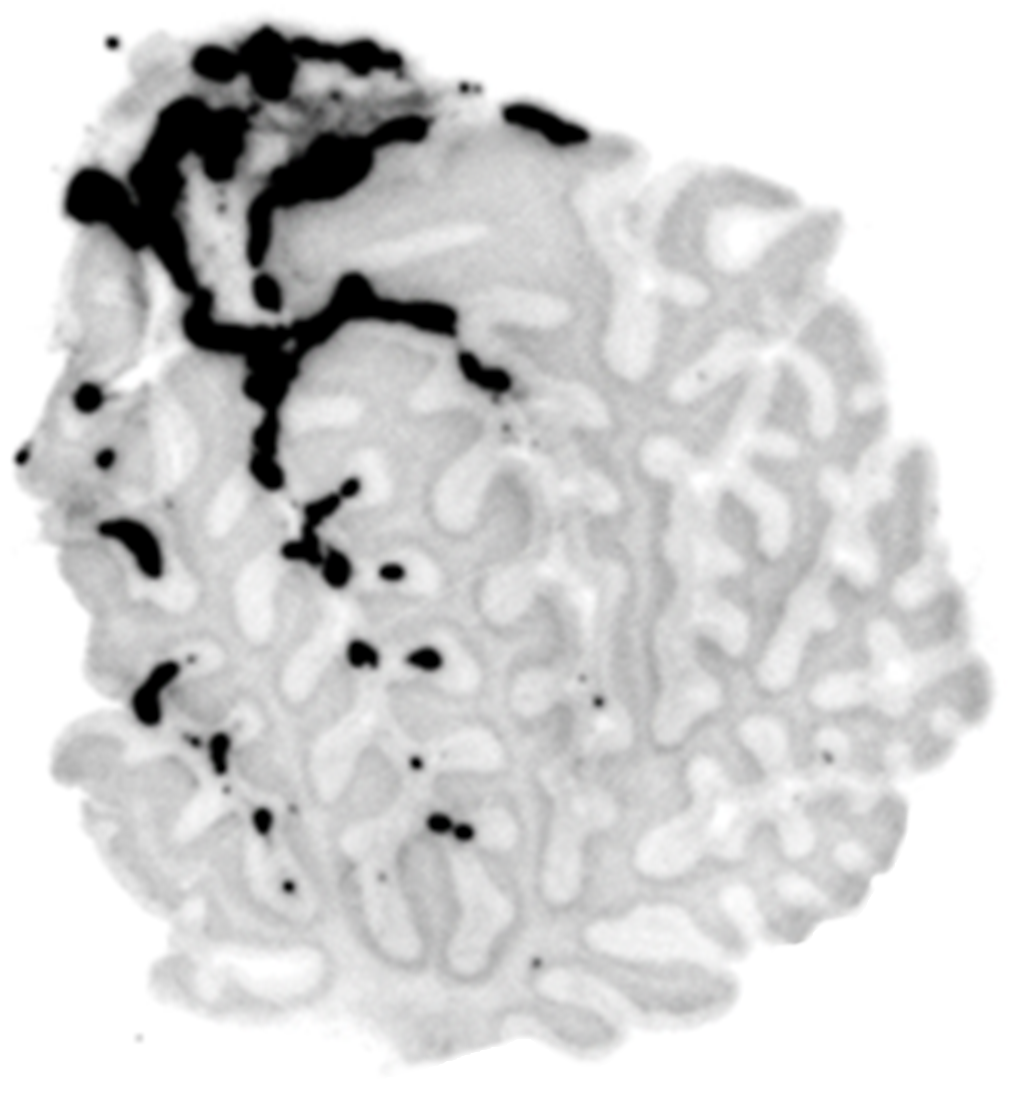


**a**

**a**

**b**

**b**

1.0 mm

**[^18^F]PI-2620 ARG**

**Supplemental Figure 9. Correlation between [^18^F]PI-2620 autoradiographic signal in the vermal leptomeninges and a) melanocytes and b) iron deposits.** Zoomed-in histological sections depict a) significantly elevated autoradiographic signal and corresponding abundance of melanocytes, and b) minimal elevated autoradiographic signal and corresponding iron deposits due to antemortem subarachnoid haemorrhage using Prussian blue staining.

**References**

1. Willroider, M., et al., *Superiority of Formalin-Fixed Paraffin-Embedded Brain Tissue for in vitro Assessment of Progressive Supranuclear Palsy Tau Pathology With [ (18) F]PI-2620.* Front Neurol, 2021. **12**: p. 684523.

2. Leuzy, A., et al., *Harmonizing tau positron emission tomography in Alzheimer's disease: The CenTauR scale and the joint propagation model.* Alzheimers Dement, 2024. **20**(9): p. 5833-5848.

3. Brendel, M., et al., *Assessment of 18F-PI-2620 as a Biomarker in Progressive Supranuclear Palsy.* JAMA Neurol, 2020. **77**(11): p. 1408-1419.
