# Supplementary material for: Exploring the origins of frequent tau-PET signal in vermal and adjacent regions
Source: Eur J Nucl Med Mol Imaging. 2025 Mar 18;52(10):3519–33. doi: 10.1007/s00259-025-07199-x (PMC12316827; doi:10.1007/s00259-025-07199-x)
Supplement: Supplementary file 2 — (DOCX 27.0 KB) [file 259_2025_7199_MOESM2_ESM.docx]

**Supplemental Table 1. Overview on samples of the PET to autopsy cohort [1].**

| **Case** | **Demographics** | | | | | **Diagnosis** | | **Autopsy determinants** | | | **Copathology** | | | | |
| --- | --- | --- | --- | --- | --- | --- | --- | --- | --- | --- | --- | --- | --- | --- | --- |
|  | Gender | Age at PET (y) | Age at death (y) | Disease  Duration (y) | Cause of death (death certificate) | Clinical  diagnosis | Autopsy  diagnosis | Brain  weight (g) | Postmortem  delay (h) | Fixation  time (d) | Aβ/α-syn/TDP-43/FUS | Frontal  cortex | Putamen | Globus pallidus | APOE |
| #1 | male | 70 | 71 | 6 | Atypical PD/  recurrent pneumonia | PSP | PSP | 1570 | 39 | 63 |  | β-Amyloid | n.a. | n.a. | n.a. |
| #2 | male | 63 | 67 | 4 | n.a. | nfPPA | PSP, AD (B&B 3), mild AGD | 1512 | 30 | 48 |  | - | n.a. | n.a. | n.a. |
| #3 | female | 75 | 75 | 6 | Loss of food and fluids | bvFTD (TBK1-mutation) | FTLD-TDP (Type A),  Lewy body disease (Braak 5, neocortical), argyrophilic grain disease (AGD), ARTAG, central pontine myelinolysis, hippocampal sclerosis, PART (B&B 2), multiple microinfarcts | 1080 | 18 | 40 |  | Synuclein, tau | tau | tau | n.a. |
| #4 | female | 73 | 77 | 6 | Cardiac arrhythmia | PSP-RS | PSP, AGD, ARTAG, subdural hemorrhage, AD (B&B 1) | 1170 | 25 | 71 |  | β-Amyloid | n.a. | n.a. | n.a. |
| #5 | male | 64 | 66 | 4 | n.a. | PSP-RS | PSP, ARTAG, intranuclear inclusions of unclear etiology, discrete TDP-43 in the brainstem | 1363 | 90 | 31 |  | - | n.a. | n.a. | n.a. |
| #6 | male | 72 | 75 | 7 | Hearth failure, COVID-19 | bvFTD/nfvPPA | PSP | 1262 | 34-58 | 124 |  | n.a. | n.a. | n.a. | n.a. |
| #7 | female | 68 | 70 | 6 | Dysphagia | PSP-RS | PSP, CAA, AD (B&B 3), AGD | 1182 | 42 | 76 |  | β-Amyloid | β-Amyloid | n.a. | n.a. |
| #8 | female | 65 | 65 | 1 | ALS-FTD | ALS-FTD, PPA | FTLD/MND-TDP, AD (B&B 3), AGD | 1261 | 48 | 88 |  | - | n.a. | n.a. | n.a. |
| #9 | female | 70 | 71 | n.a. | n.a. | CBS | n.a. | n.a. | n.a. | n.a. |  | n.a. | n.a. | n.a. | n.a. |

**Supplemental Table 1.** Abbreviations: y = years; PD = Parkinson’s disease; ALS = amyotrophic lateral sclerosis; FTD = frontotemporal dementia; PSP-RS = progressive supranuclear palsy Richardson syndrome; nf = non-fluent; PPA = primary progressive aphasia; bv = behavioral variant; AD = Alzheimer’s disease; CAA = cerebral amyloid angiopathy; CBS = corticobasal syndrome; AGD = agyrophilic grain disease; ARTAG = aging-related tau astrogliopathy; B&B = Braak and Braak; TDP-43 = TAR DNA-binding protein 43; MND = motor neuron disease; PART = primary age-related tauopathy; Aβ = β-amyloid; APOE = apolipoprotein E; α-syn = alphasynuclein; n.a. = not available; „-„ = negative; co-pathology: FUS not examined.

**Supplemental Table 2.** **Sex-related differences of vermal [^18^F]PI-2620 SUVR and DVR.**

| **Cohort** | **Sex** | **n** | **SUVR_Ver/Cbl_** | **p** | **F** | **p*** | **F*** | **DVR** | **p** | **F** | **p*** | **F*** |
| --- | --- | --- | --- | --- | --- | --- | --- | --- | --- | --- | --- | --- |
| **All** | Female | 121 | 1.18 ± 0.23 | 0.058 | 3.638 | 0.056 | 3.686 | 0.88 ± 0.10 | <0.0001 | 25.057 | <0.0001 | 25.072 |
|  | Male | 153 | 1.24 ± 0.32 |  |  |  |  | 0.95 ± 0.13 |  |  |  |  |
| **AD** | Female | 44 | 1.15 ± 0.23 | 0.009 | 7.170 | 0.009 | 7.086 | 0.85 ± 0.08 | <0.0001 | 28.007 | <0.0001 | 27.666 |
|  | Male | 41 | 1.30 ± 0.30 |  |  |  |  | 0.96 ± 0.12 |  |  |  |  |
| **4R** | Female | 59 | 1.16 ± 0.21 | 0.186 | 1.764 | 0.196 | 1.688 | 0.90 ± 0.11 | 0.015 | 6.053 | 0.017 | 5.835 |
|  | Male | 88 | 1.23 ± 0.34 |  |  |  |  | 0.96 ± 0.14 |  |  |  |  |
| **DC** | Female | 8 | 1.35 ± 0.25 | 0.124 | 2.558 | 0.216 | 1.630 | 0.94 ± 0.11 | 0.744 | 0.109 | 0.959 | 0.003 |
|  | Male | 16 | 1.14 ± 0.32 |  |  |  |  | 0.92 ± 0.13 |  |  |  |  |
| **HC** | Female | 10 | 1.26 ± 0.29 | 0.641 | 0.227 | 0.847 | 0.038 | 0.87 ± 0.07 | 0.012 | 8.000 | 0.136 | 2.482 |
|  | Male | 8 | 1.32 ± 0.18 |  |  |  |  | 0.95 ± 0.04 |  |  |  |  |

**Supplemental Table 2.** Sex differences in vermal tau-PET, assessed using Analysis of Variance with age (*) as a covariate.

**Supplemental References**

1. Slemann, L., et al., *Neuronal and oligodendroglial, but not astroglial, tau translates to in vivo tau PET signals in individuals with primary tauopathies.* Acta Neuropathologica, 2024. **148**(1): p. 70.
